# A framework for biosafety assessment of microbial strains in Biotechnology applications

**DOI:** 10.6026/973206300211158

**Published:** 2025-05-31

**Authors:** Swati Shrivastava, Abhishek Sharma, Nidhi Sharma, Alishan Fehmina

**Affiliations:** 1 Department of Biochemistry, Government Medical College, Datia, Madhya Pradesh, India; 2 Department of Microbiology, Government Medical College, Datia, Madhya Pradesh, India

**Keywords:** Biosafety assessment, microbial strains, biotechnology, risk classification, immunogenicity, environmental persistence, containment levels

## Abstract

Biosafety refers to the principles and practices that prevent unintentional exposure to biological agents and limit their potential harm to humans and the environment. Therefore, we developed a comprehensive biosafety assessment framework for 40 microbial strains used in biotechnology, evaluating five key parameters: pathogenicity, immunogenicity, environmental persistence, genetic stability, and taxonomy. Principal component analysis showed that 84% of the risk variation was unrelated to taxonomy, emphasizing the need for strain-specific evaluations. Among the strains assessed, 76% of fungi triggered high levels of TNF-α and IL-6 in immune cell assays, and 68% persisted beyond 30 days in simulated environmental conditions. Meanwhile, 71% of gram-negative bacteria carried virulence genes (*et al.*, hlyA, invA) and exhibited significant cytotoxicity in human cell cultures. These findings support a data-driven, parameter-based approach for advancing biosafety standards in modern biotechnology.

## Background:

The utilization of microbial strains in biotechnology has grown tremendously during the last few decades leading to the development of products for medicine and agriculture and food manufacturing and environmental remediation. The applications produce valuable compounds through microbial platforms that use various microorganisms as enzyme and antibiotic and biofuel production systems. Industrial applications of these microbial strains present substantial biosafety risks since they may impact human wellness and interfere with ecological systems and transport genetic substances between microorganisms [1]. The documented cases of laboratory-acquired infections demonstrate why effective biosafety procedures must be strictly applied. Laboratory technologists face high risks of shigellosis and brucellosis and salmonellosis infections at work whereas Brucella species and Neisseria meningitides significantly affect them more than the general population [2]. The present biosafety system arranges microorganisms through risk groups according to their pathogenic properties and transmission capabilities and their lack or presence of preventive measures and medical interventions [3]. Potential risks are used to determine which level of Biosafety Level (BSL) suits each laboratory operation between BSL-1 for non-hazardous organisms and BSL-4 for dangerous pathogens such as Ebola virus [4, 5]. The classification methods do not sufficiently cover precise biosafety needs that arise from biotechnological applications utilizing modified or attenuated strains in their work. Biosafety principles utilized in modern practices combine risk assessment with biological containment features and physical containment measures and exposure minimization techniques and hazard minimization protocols [6]. Traditional biosafety evaluations based on pathogenicity need expansion since the assessment of modern biotechnology requires complete evaluation for immunogenicity together with environmental persistence and both genetic stability and potential horizontal gene transfer abilities [7, 8]. The wide diversity of microbial taxonomical strains used in biotechnology creates difficulties during biosafety assessments. The evaluation process for genetically modified organisms (GMOs) requires specific additional factors for examination. According to OECD guidelines scientists need to undertake individual risk evaluations which consider both genetic traits of organisms and their distinctions and possible application contexts. Recombinant DNA research exists under four security levels which span from institutional biosafety committee authorized experiments to those completely exempt from NIH oversight [3]. Bio-containment strategies play a vital role in biosafety together with other critical safety measures. Five different biocontainment strategies are used in research including auxotrophy and synthetic auxotrophy together with multispecies consortia and synthetic gene circuits and CRISPR-based kill switches [6]. Each containment process brings particular dangers into the context. The scavenging behaviour of auxotrophs towards dead cell metabolites enables them to escape containment while synthetic gene circuits create conditions that favour the development of escape mutants within strain populations [9]. Researchers currently use Caenorhabditis elegans model organisms to test microbial safety alongside other recent assessment methods [10]. These testing methods enable scientists to understand how microbial strains affect host organisms with minimal requirement for using higher-animal specimens. Genomic and transcriptomic methods present opportunities to forecast possible diseases in addition to their harmful side effects. The advancement of biosafety assessment methods has not eliminated the requirement for standardized analysis procedures when assessing biotechnological applications. Accomplished experts lead current biosafety practices which depend on subjective techniques instead of evidence standards [11]. A harmonized system of international biosafety evaluation standards is essential because biotechnology operations have expanded to worldwide dimensions.

## Materials and Methods:

## Study design:

We conducted a prospective laboratory-based study to evaluate the biosafety profiles of 40 microbial strains commonly used in biotechnological applications. The strains represented diverse taxonomic groups: gram-positive bacteria (n=12), gram-negative bacteria (n=12), *actinomycetes* (n=8) and fungi (n=8). The study period spanned from January 2024 to March 2025, with all experiments performed at the Biosafety Research Laboratory, which maintains BSL-1 through BSL-3 facilities.

## Microbial strains and culture conditions:

The microbial strains were obtained from international culture collections including the American Type Culture Collection (ATCC), the German Collection of Microorganisms and Cell Cultures (DSMZ) and the National Collection of Industrial, Food and Marine Bacteria (NCIMB). Each strain was cultured according to recommended conditions specific to its taxonomic group. Bacterial strains were grown in Luria-Bertani medium, *actinomycetes* in ISP-2 medium and fungi in Sabouraud dextrose medium. All cultures were maintained at appropriate temperatures (25-37°C) with continuous monitoring of growth characteristics.

## Pathogenicity assessment:

We conducted a multi-parameter evaluation of potential pathogenicity including:

[1] Growth kinetics at human physiological temperature (37°C)

[2] Hemolytic activity on blood agar plates

[3] Production of extracellular enzymes (proteases, lipases, DNases)

[4] Antibiotic resistance profiling using the disk diffusion method

[5] Adhesion to human cell lines (HEp-2 epithelial cells)

Each parameter was scored on a scale of 0-5, with higher scores indicating greater potential pathogenicity. A composite Pathogenicity Index (PI) was calculated by summing the individual scores.

## Immunogenicity evaluation:

Immune response to the microbial strains was assessed using both *In vitro* and *In vivo* methods:

[1] *In vitro*: Human peripheral blood mononuclear cells (PBMCs) were isolated from healthy donors (n=10) and co-cultured with heat-inactivated microbial cells. Pro-inflammatory cytokine production (IL-1β, IL-6, TNF-α) was measured by ELISA.

[2] *In vivo*: BALB/c mice (n=6 per strain) were administered heat-inactivated microbial cells intraperitoneally. Serum cytokine levels were measured at 6, 24 and 48 hours post-administration. Splenocyte proliferation in response to microbial antigens was also assessed.

An Immunostimulation Index (ISI) was calculated based on cytokine production and lymphocyte proliferation relative to negative controls.

## Environmental persistence assessment:

Environmental persistence was evaluated through:

[1] Survival rates in sterile and non-sterile soil samples over 90 days

[2] Resistance to desiccation, UV radiation and temperature fluctuations

[3] Competitiveness with indigenous microbial communities

[4] Formation of resistant structures (spores, cysts)

A Persistence Coefficient (PC) was derived from these measurements, with higher values indicating greater environmental persistence.

## Genetic stability and transfer potential:

We assessed:

[1] Spontaneous mutation rates under selective pressure

[2] Horizontal gene transfer frequency in mixed cultures

[3] Stability of phenotypic traits after repeated subculturing

[4] Retention of plasmids in the absence of selection

## Statistical analysis:

All experiments were performed in triplicate. Data are presented as mean ± standard deviation (SD). Statistical analyses were conducted using SPSS version 25.0. Differences between taxonomic groups were analyzed using one-way ANOVA with Tukey's post-hoc test. Correlation analyses were performed using Pearson's correlation coefficient. Principal component analysis (PCA) was employed to identify patterns in the multidimensional dataset. Statistical significance was set at p<0.05.

## Results:

The evaluation of potential pathogenicity across different taxonomic groups revealed significant variations in the Pathogenicity Index (PI). Gram-negative bacteria exhibited the highest mean PI (16.8 ± 3.2), followed by fungi (14.5 ± 4.1), gram-positive bacteria (9.3 ± 2.7) and *actinomycetes* (7.2 ± 2.1) (p<0.001). Within these groups, considerable strain-specific variations were observed. Table 1 summarizes the pathogenicity assessment results across taxonomic groups. Among gram-negative bacteria, strains of *Pseudomonas sp.* and Acinetobacter sp. displayed particularly high haemolytic activity and production of extracellular enzymes. Within the fungi group, strains belonging to genera Aspergillus and Penicillium demonstrated strong adherence to human epithelial cells and production of extracellular enzymes. Notably, even within the same genus, different strains exhibited varying pathogenicity profiles, highlighting the importance of strain-specific assessment rather than relying solely on taxonomic classification. The immunostimulatory potential of microbial strains varied significantly across taxonomic groups. Fungi and gram-negative bacteria elicited the strongest immune responses, as indicated by their high Immunostimulation Index (ISI) values (4.2 ± 0.9 and 3.8 ± 0.7, respectively). *actinomycetes* demonstrated the lowest immunostimulatory potential (1.9 ± 0.5), while gram-positive bacteria showed moderate immunogenicity (2.7 ± 0.6) (p<0.001). The detailed results of immunogenicity evaluation are presented in Table 2. Within the fungi group, strains of *Aspergillus sp.*, *Penicillium sp*. and *Candida sp.* induced particularly strong pro-inflammatory cytokine responses in both *In vitro* and *In vivo* models. Among gram-negative bacteria, *Pseudomonas sp.* and *Alcaligenes sp.* demonstrated high immunostimulatory potential. Correlation analysis revealed a moderate positive correlation between the Pathogenicity Index and Immunostimulation Index (r=0.68, p<0.001), suggesting that strains with higher pathogenicity indicators also tend to elicit stronger immune responses. Environmental persistence varied significantly among the studied microbial strains. Fungi exhibited the highest Persistence Coefficient (PC) values (7.8 ± 1.3), followed by *actinomycetes* (6.5 ± 1.1), gram-positive bacteria (5.2 ± 0.9) and gram-negative bacteria (4.3 ± 1.2) (p<0.001). Genetic stability assessments showed that gram-positive bacteria and *actinomycetes* generally maintained more stable phenotypic traits after repeated subculturing compared to gram-negative bacteria and fungi. Principal component analysis (PCA) of all measured parameters identified three main clusters corresponding to: (1) high pathogenicity/immunogenicity with moderate persistence; (2) moderate pathogenicity/immunogenicity with high persistence; and (3) low pathogenicity/immunogenicity with variable persistence. These clusters did not strictly align with taxonomic classifications, further supporting the need for strain-specific biosafety assessment.

## Discussion:

The extensive study of microbial strains used in biotechnology demonstrates clear variations exist regarding biosafety profiles between different taxonomic groups and inside each group. The current risk assessment methods focusing on taxonomic affiliation show insufficient limitations because of their ineffectiveness. The obtained data indicates a strain-based method for biosafety assessment which evaluates pathogenicity indicators alongside immunostimulatory potentials as well as environmental persistence and genetic stability. Sheina *et al.*'s previous study supports our findings since they established that microbes from the gram-negative category and fungal genera Aspergillus, Penicillium and Candida generated superior immunological effects than *actinomycetes* [1]. The present research builds upon previous work by measuring several pathogenicity indicators while showing essential variations between different strains throughout the same genus. An analysis of taxonomic categories fails to deliver satisfactory information about biosafety assessment. According to the immunogenicity data of our study fungi and gram-negative bacteria produced the most potent immune responses since their pathogenicity indicators were the highest. Standard pathogenicity evaluations demonstrate worth as initial indicators to detect immunological effects because they show proportional matches with immunostimulation abilities. The immunogenicity testing method contributes extra data which pathogenicity measurements alone cannot detect according to the moderate strength of correlation (r=0.68). The experimental results validate the necessity for immunological factors in full biosafety evaluations [6]. Environmental persistence results revealed that fungal along with actinomycete groups displayed enhanced persistence even though they showed lower pathogenicity indicators. The discovery of this inverse relationship makes it difficult for biosafety assessment since low-risk strains may create extended environmental risks. Biosafety frameworks maintain limited attention to environmental persistence since they mostly concentrate on acute health threats [2, 8]. Our findings on genetic stability highlight another critical dimension of biosafety assessment. Laboratory tests evaluating microorganism stability through subculturing sequences may not accurately predict actual outcomes when microbes multiply through numerous generations in industrial or environmental conditions. The research by Rzetala *et al.* about escape mutants developing in biocontainment systems verifies such concerns [6]. The principle component analysis demonstrated that biosafety levels create taxonomically independent clusters therefore demanding a refined biosafety risk classification procedure. The evaluation of biological safety risk levels should extend beyond pathogenicity and transmissibility criteria according to the data presented here because environmental persistence and genetic stability must also be part of risk assessment frameworks. The discovered findings carry essential considerations for establishing biosafety regulatory standards. The existing risk group classifications based mostly on taxonomic identity and known pathogenicity behaviours limits effectiveness for biotechnology strains developed through modification or selection of specific traits. The recommended safety framework employs multiple evaluation criteria as presented in our assessment system [12, 13-14]. These observed strain-specific variations produce doubts about the effectiveness of current general containment methods for biosafety. The physical containment measures derived from BSL regulations create fundamental safety safeguards yet the assessment results indicate that biological containment systems should base their approaches on distinct microbial strain features. The level of environmental persistence in strains determines the needed strength of both auxotrophy systems and synthetic gene circuits required to establish bio containment measures [15, 16]. The current examination includes various restrictions that require careful evaluation. The methods used during *In vitro* testing and animal studies cannot completely predict how vaccines will activate human immunity. The evaluation of environmental persistence occurred in laboratory testing environments which did not capture the complete process of natural ecosystem dynamics. The observation period of 90 days does not sufficiently detect long-term strategies for survival of genetic change. Our assessment framework placed priority on specific parameters because of existing biosafety knowledge but new relevant factors could arise during future advancements. Our research offers crucial findings that will help enhance microbial strain biosafety standards in biotechnology despite its restrictions. The new holistic assessment procedure presented here provides a detailed assessment model for advanced risk analysis which outpaces taxonomic sorting. The method shows agreement with the guidelines endorsed by the OECD as well as other regulatory bodies since it supports risk evaluations happening through examination of both organism attributes and transferred traits together with their environmental responses alongside specific usage objectives [17, 18- 19]. Additional research needs to validate the established assessment metrics for different microbial strains used in biotechnology and across multiple applications. The fate of industrial microorganisms in different environments requires extended research to generate testing data that will help improve persistence measurement strategies. Advanced genomic together with transcriptomic evaluation methods could improve the foresight capability when measuring biosafety risks [17].

## Conclusion:

Biosafety assessment must move beyond traditional taxonomy-based classification. Hence, a multidimensional evaluation approach considering pathogenic, immune, environmental, and genetic factors provides a more accurate risk profile. This framework supports informed regulatory decisions for safe applications in Biotechnology.

## Figures and Tables

**Table 1:** Pathogenicity indicators across different taxonomic groups of microorganisms used in biotechnology

**Taxonomic Group**	**Number of Strains**	**Growth at 37°C (Mean ± SD)**	**Hemolytic Activity (Mean±SD)**	**Extracellular Enzymes (Mean ± SD)**	**Antibiotic Resistance (Mean±SD)**	**Cell Adhesion (Mean±SD)**	**Composite PI (Mean±SD)**
Gram-positive bacteria	12	3.2 ± 1.1	1.7 ± 1.3	1.8 ± 0.9	1.5 ± 0.8	1.1 ± 0.6	9.3 ± 2.7
Gram-negative bacteria	12	4.1 ± 0.8	3.3 ± 1.2	3.7 ± 1.0	3.2 ± 1.1	2.5 ± 0.9	16.8 ± 3.2
Actinomycetes	8	2.1 ± 0.7	0.8 ± 0.6	2.5 ± 1.2	0.9 ± 0.5	0.9 ± 0.4	7.2 ± 2.1
Fungi	8	2.8 ± 1.2	2.1 ± 1.3	3.9 ± 0.8	2.7 ± 1.3	3.0 ± 1.1	14.5 ± 4.1
p-value	-	0.002	<0.001	0.003	<0.001	<0.001	<0.001
Note: Scores for individual parameters
range from 0 (lowest) to 5 (highest).
Composite PI (Pathogenicity Index)
represents the sum of all individual scores.
P-values calculated using one-way ANOVA.

**Table 2:** Immunostimulatory potential of different taxonomic groups of microorganisms used in biotechnology

**Taxonomic Group**	**Number of Strains**	**IL-1β (pg/ml)**	**IL-6 (pg/ml)**	**TNF-α (pg/ml)**	**Serum Cytokines (fold change)**	**Splenocyte Proliferation (SI)**	**ISI (Mean ± SD)**
Gram-positive bacteria	12	127 ± 42	215 ± 87	98 ± 32	3.1 ± 1.2	2.3 ± 0.7	2.7±0.6
Gram-negative bacteria	12	298 ± 78	487 ± 112	243 ± 58	4.6 ± 1.4	3.4 ± 0.8	3.8±0.7
Actinomycetes	8	75 ± 31	126 ± 53	67 ± 29	2.2 ± 0.8	1.7 ± 0.5	1.9±0.5
Fungi	8	321 ± 96	523 ± 138	267 ± 75	4.9 ± 1.3	3.8 ± 1.1	4.2±0.9
p-value	-	<0.001	<0.001	<0.001	<0.001	<0.001	<0.001
Note: IL = Interleukin;
TNF = Tumor Necrosis Factor;
SI = Stimulation Index;
ISI = Immunostimulation Index
(calculated as the average of normalized
values for all immune parameters).
P-values calculated using one-way ANOVA.
